# Chlorido(10,11,12,13-tetra­hydro-4,5,9,14-tetra­aza­benzo[*b*]triphenyl­ene-κ^2^ *N* ^4^,*N* ^5^)copper(I)

**DOI:** 10.1107/S1600536811034817

**Published:** 2011-08-31

**Authors:** Jun Hong, Xiu-Ying Li, Seik Weng Ng

**Affiliations:** a Department of Chemistry, Jilin Normal University, Siping 136000, People’s Republic of China; b Department of Chemistry, University of Malaya, 50603 Kuala Lumpur, Malaysia; c Chemistry Department, King Abdulaziz University, PO Box 80203 Jeddah, Saudi Arabia

## Abstract

The Cu^I^ atom in the title compound, [CuCl(C_18_H_14_N_4_)], is *N*,*N*′-chelated by the *N*-heterocyclic ligand and coordinated by one Cl^−^ anion in a distorted trigonal geometry. In the crystal, the Cu^I^ atom is disordered over two positions in a 0.667 (6):0.333 (6) ratio. The deviation of the Cu atom from the N/N/Cl coordination plane is 0.013 (3) Å for the major component and 0.073 (6) Å for the minor component. Two methyl­ene C atoms are also disordered over two positions in a 0.667 (6):0.333 (6) ratio.

## Related literature

For the synthesis of the *N*-heterocyclic ligand, see: Che *et al.* (2006 ▶).
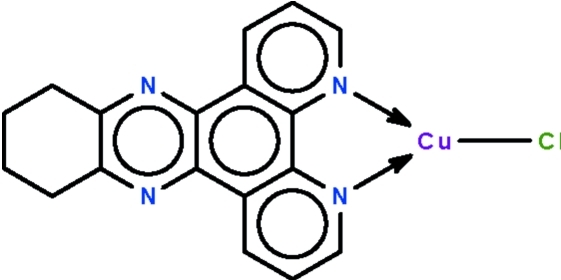


## Experimental

### 

#### Crystal data

[CuCl(C_18_H_14_N_4_)]
*M* *_r_* = 385.32
Monoclinic, 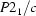

*a* = 7.9405 (10) Å
*b* = 15.8861 (19) Å
*c* = 12.6312 (16) Å
β = 99.531 (2)°
*V* = 1571.4 (3) Å^3^
*Z* = 4
Mo *K*α radiation
μ = 1.57 mm^−1^
*T* = 293 K
0.15 × 0.10 × 0.10 mm

#### Data collection

Bruker SMART APEX diffractometer
Absorption correction: multi-scan (*SADABS*; Sheldrick, 1996 ▶) *T*_min_ = 0.799, *T*_max_ = 0.859
8464 measured reflections
3064 independent reflections
1957 reflections with *I* > 2σ(*I*)
*R*_int_ = 0.036

#### Refinement

*R*[*F* ^2^ > 2σ(*F* ^2^)] = 0.042
*wR*(*F* ^2^) = 0.119
*S* = 0.99
3064 reflections
233 parameters
16 restraints
H-atom parameters constrained
Δρ_max_ = 0.26 e Å^−3^
Δρ_min_ = −0.34 e Å^−3^

### 

Data collection: *APEX2* (Bruker, 2002 ▶); cell refinement: *SAINT* (Bruker, 2002 ▶); data reduction: *SAINT*; program(s) used to solve structure: *SHELXS97* (Sheldrick, 2008 ▶); program(s) used to refine structure: *SHELXL97* (Sheldrick, 2008 ▶); molecular graphics: *X-SEED* (Barbour, 2001 ▶); software used to prepare material for publication: *publCIF* (Westrip, 2010 ▶).

## Supplementary Material

Crystal structure: contains datablock(s) global, I. DOI: 10.1107/S1600536811034817/xu5308sup1.cif

Structure factors: contains datablock(s) I. DOI: 10.1107/S1600536811034817/xu5308Isup2.hkl

Additional supplementary materials:  crystallographic information; 3D view; checkCIF report

## Figures and Tables

**Table 1:** Selected bond lengths (Å)

Cu1—Cl1	2.102 (3)
Cu1—N1	1.928 (4)
Cu1—N2	2.236 (4)
Cu1′—Cl1	2.112 (6)
Cu1′—N1	2.268 (7)
Cu1′—N2	1.858 (6)

**Fig. 1.**
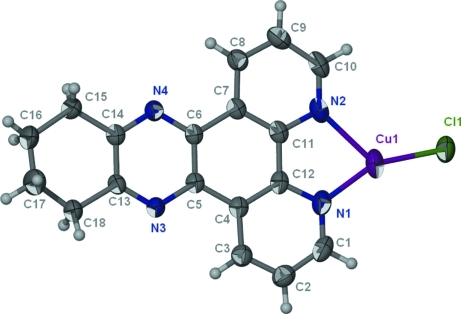
Thermal ellipsoid plot (Barbour, 2001) of CuCl(C18H14N4) at the 50% probability level; hydrogen atoms are drawn as spheres of arbitrary radius. The disorder is not shown.

## supplementary crystallographic information

### Comment

There is extensive literature on the chelating ability of 1,10-phenanthroline and its derivatives. The *N*-heterocycle, 10,11,12,13-tetrahydro-4,5,9,14-tetraazabenzo[*b*]triphenylene, is a new addition to this class of chelates. It reacts with copper(II) chloride under hydrothermal conditions to yield a 1:1 copper(I) chloride adduct (Scheme I). The Cu^I^ atom is *N*,*N*'-chelated by the *N*-heterocycle, and it shows trigonal coordination. The atom lies on the trigonal plane defined by the two chelating N atoms and the chlorine atom (Fig. 1). The aromatic ring system is planar; however, the *N*-heterocycle is buckled in the region of the cyclohexene part owing to the *sp*^3^ nature of the methylene C atoms.

### Experimental

The *N*-heterocycle was synthesized according to a literature method (Che *et al.*, 2006). To a copper(II) chloride dihydrate (0.5 mmol), the ligand (0.5 mmol) and water (5 ml) was added triethylamine to a final pH of 5.5. The mixture was heated in a Teflon-lined, stainless-steel Paar bomb at 423 K for 3 days. The Parr bomb was then cooled slowly; yellow blocks were isolated by hand.

### Refinement

Carbon-bound H-atoms were placed in calculated positions (C—H 0.95 to 0.97 Å) and were included in the refinement in the riding model approximation, with *U*(H) set to 1.2 to 1.5*U*(C).

The copper atom is disordered over two positions in an approximate 2:1 ratio. The cyclohexene portion of the *N*-heterocycle is disordered with respect to the two methylene C atoms that are not directly connected to an aromatic C atom. For this cyclohexene portion, 1,2-related distances involving all four methylene C atoms were restrained to 1.54±0.01 Å and the 1,3-related ones to 2.51±0.01 Å. The temperature factors of the primed atoms (C16', C17') were set to those of the unprimed ones. The disorder refined to nearly 2:1. As such, the occupancy of the primed C atoms were regarded as that of the Cu1' atom. The disorder then refined to 66.8 (6): 33.2.

### Crystal data

**Table:**

[CuCl(C_18_H_14_N_4_)]	*F*(000) = 784
*M**_r_* = 385.32	*D*_x_ = 1.629 Mg m^−^^3^
Monoclinic, *P*2_1_/*c*	Mo *K*α radiation, λ = 0.71073 Å
Hall symbol: -P 2ybc	Cell parameters from 1330 reflections
*a* = 7.9405 (10) Å	θ = 2.6–21.7°
*b* = 15.8861 (19) Å	µ = 1.57 mm^−^^1^
*c* = 12.6312 (16) Å	*T* = 293 K
β = 99.531 (2)°	Prism, yellow
*V* = 1571.4 (3) Å^3^	0.15 × 0.10 × 0.10 mm
*Z* = 4	

### Data collection

**Table:**

Bruker SMART APEX diffractometer	3064 independent reflections
Radiation source: fine-focus sealed tube	1957 reflections with *I* > 2σ(*I*)
graphite	*R*_int_ = 0.036
ω scans	θ_max_ = 26.0°, θ_min_ = 2.1°
Absorption correction: multi-scan (*SADABS*; Sheldrick, 1996)	*h* = −9→9
*T*_min_ = 0.799, *T*_max_ = 0.859	*k* = −19→11
8464 measured reflections	*l* = −15→15

### Refinement

**Table:**

Refinement on *F*^2^	Primary atom site location: structure-invariant direct methods
Least-squares matrix: full	Secondary atom site location: difference Fourier map
*R*[*F*^2^ > 2σ(*F*^2^)] = 0.042	Hydrogen site location: inferred from neighbouring sites
*wR*(*F*^2^) = 0.119	H-atom parameters constrained
*S* = 0.99	*w* = 1/[σ^2^(*F*_o_^2^) + (0.0591*P*)^2^] where *P* = (*F*_o_^2^ + 2*F*_c_^2^)/3
3064 reflections	(Δ/σ)_max_ = 0.001
233 parameters	Δρ_max_ = 0.26 e Å^−^^3^
16 restraints	Δρ_min_ = −0.34 e Å^−^^3^

### Fractional atomic coordinates and isotropic or equivalent isotropic displacement parameters (Å^2^)

**Table:**

	*x*	*y*	*z*	*U*_iso_*/*U*_eq_	Occ. (<1)
Cu1	0.5949 (4)	0.27795 (17)	0.5683 (2)	0.0588 (5)	0.667 (6)
Cu1'	0.6063 (7)	0.2766 (4)	0.5259 (5)	0.0618 (10)	0.333 (6)
Cl1	0.54512 (13)	0.14874 (5)	0.54499 (7)	0.0650 (3)	
N1	0.6098 (3)	0.38256 (15)	0.6466 (2)	0.0497 (7)	
N2	0.6947 (3)	0.36198 (16)	0.4508 (2)	0.0500 (7)	
N3	0.7722 (3)	0.67671 (15)	0.64582 (18)	0.0409 (6)	
N4	0.8683 (3)	0.65335 (15)	0.44444 (18)	0.0411 (6)	
C1	0.5643 (4)	0.3927 (2)	0.7432 (3)	0.0567 (9)	
H1	0.5231	0.3463	0.7759	0.068*	
C2	0.5758 (4)	0.4695 (2)	0.7973 (3)	0.0538 (8)	
H2	0.5426	0.4738	0.8643	0.065*	
C3	0.6361 (4)	0.5382 (2)	0.7512 (2)	0.0479 (8)	
H3	0.6440	0.5898	0.7862	0.058*	
C4	0.6863 (4)	0.53036 (18)	0.6501 (2)	0.0392 (7)	
C5	0.7535 (3)	0.60072 (18)	0.5960 (2)	0.0381 (7)	
C6	0.8016 (3)	0.58937 (18)	0.4953 (2)	0.0365 (7)	
C7	0.7847 (3)	0.50718 (18)	0.4442 (2)	0.0392 (7)	
C8	0.8325 (4)	0.49183 (19)	0.3433 (2)	0.0478 (8)	
H8	0.8795	0.5347	0.3073	0.057*	
C9	0.8091 (4)	0.4130 (2)	0.2986 (3)	0.0560 (9)	
H9	0.8403	0.4016	0.2322	0.067*	
C10	0.7377 (4)	0.3504 (2)	0.3545 (3)	0.0584 (9)	
H10	0.7190	0.2977	0.3228	0.070*	
C11	0.7182 (4)	0.43948 (18)	0.4953 (2)	0.0416 (7)	
C12	0.6714 (4)	0.45138 (18)	0.6012 (2)	0.0410 (7)	
C13	0.8372 (4)	0.73873 (17)	0.5965 (2)	0.0396 (7)	
C14	0.8849 (4)	0.72754 (17)	0.4930 (2)	0.0399 (7)	
C15	0.9599 (4)	0.79779 (18)	0.4360 (2)	0.0517 (8)	
H15A	0.8991	0.8009	0.3629	0.062*	0.667 (6)
H15B	1.0781	0.7843	0.4327	0.062*	0.667 (6)
H15C	0.8721	0.8206	0.3810	0.062*	0.333 (6)
H15D	1.0498	0.7752	0.4008	0.062*	0.333 (6)
C16	0.9535 (7)	0.8843 (3)	0.4885 (4)	0.0508 (16)	0.667 (6)
H16A	0.8399	0.9079	0.4697	0.061*	0.667 (6)
H16B	1.0335	0.9221	0.4624	0.061*	0.667 (6)
C17	0.9997 (7)	0.8754 (3)	0.6111 (4)	0.0549 (16)	0.667 (6)
H17A	1.1104	0.8486	0.6296	0.066*	0.667 (6)
H17B	1.0059	0.9306	0.6443	0.066*	0.667 (6)
C16'	1.0331 (12)	0.8687 (5)	0.5113 (7)	0.0508 (16)	0.33
H16C	1.1404	0.8506	0.5533	0.061*	0.333 (6)
H16D	1.0559	0.9172	0.4692	0.061*	0.333 (6)
C17'	0.9110 (14)	0.8937 (4)	0.5862 (7)	0.0549 (16)	0.33
H17C	0.9632	0.9378	0.6338	0.066*	0.333 (6)
H17D	0.8080	0.9166	0.5440	0.066*	0.333 (6)
C18	0.8636 (5)	0.82230 (19)	0.6522 (2)	0.0569 (9)	
H18A	0.8972	0.8131	0.7287	0.068*	0.667 (6)
H18B	0.7565	0.8530	0.6414	0.068*	0.667 (6)
H18C	0.7592	0.8372	0.6783	0.068*	0.333 (6)
H18D	0.9525	0.8160	0.7143	0.068*	0.333 (6)

### Atomic displacement parameters (Å^2^)

**Table:**

	*U*^11^	*U*^22^	*U*^33^	*U*^12^	*U*^13^	*U*^23^
Cu1	0.0555 (6)	0.0341 (5)	0.0832 (13)	−0.0029 (4)	0.0005 (9)	0.0019 (9)
Cu1'	0.0573 (12)	0.0405 (11)	0.085 (3)	−0.0055 (8)	0.004 (2)	0.009 (2)
Cl1	0.0841 (7)	0.0418 (5)	0.0688 (6)	−0.0144 (4)	0.0114 (5)	0.0013 (4)
N1	0.0432 (15)	0.0390 (16)	0.0676 (18)	0.0012 (12)	0.0107 (14)	0.0075 (14)
N2	0.0509 (16)	0.0357 (15)	0.0606 (18)	0.0034 (12)	0.0012 (14)	−0.0063 (13)
N3	0.0453 (14)	0.0362 (14)	0.0418 (14)	−0.0020 (11)	0.0089 (11)	0.0008 (12)
N4	0.0440 (15)	0.0365 (15)	0.0424 (14)	−0.0003 (11)	0.0059 (11)	0.0005 (12)
C1	0.049 (2)	0.047 (2)	0.077 (2)	0.0021 (16)	0.0190 (18)	0.0199 (19)
C2	0.053 (2)	0.056 (2)	0.056 (2)	0.0043 (17)	0.0174 (16)	0.0070 (18)
C3	0.0487 (19)	0.0446 (19)	0.0511 (19)	−0.0014 (15)	0.0101 (15)	−0.0003 (16)
C4	0.0343 (16)	0.0392 (18)	0.0426 (18)	0.0016 (13)	0.0020 (13)	0.0017 (14)
C5	0.0357 (16)	0.0355 (16)	0.0421 (17)	0.0003 (13)	0.0033 (13)	0.0002 (14)
C6	0.0333 (15)	0.0341 (16)	0.0404 (17)	0.0011 (12)	0.0008 (13)	0.0002 (13)
C7	0.0349 (16)	0.0368 (17)	0.0431 (17)	0.0041 (13)	−0.0014 (13)	−0.0006 (14)
C8	0.055 (2)	0.0420 (19)	0.0451 (18)	0.0063 (15)	0.0041 (15)	−0.0017 (15)
C9	0.064 (2)	0.052 (2)	0.050 (2)	0.0103 (17)	0.0035 (17)	−0.0109 (17)
C10	0.065 (2)	0.039 (2)	0.066 (2)	0.0043 (17)	−0.0037 (19)	−0.0141 (18)
C11	0.0380 (16)	0.0367 (17)	0.0467 (18)	0.0047 (13)	−0.0025 (14)	−0.0018 (15)
C12	0.0320 (15)	0.0346 (17)	0.0549 (19)	0.0017 (13)	0.0029 (14)	0.0049 (15)
C13	0.0419 (17)	0.0336 (16)	0.0430 (18)	−0.0013 (13)	0.0064 (14)	−0.0040 (14)
C14	0.0381 (17)	0.0368 (18)	0.0432 (17)	0.0001 (13)	0.0023 (13)	0.0006 (14)
C15	0.063 (2)	0.045 (2)	0.0504 (19)	−0.0068 (16)	0.0176 (16)	0.0015 (16)
C16	0.062 (4)	0.033 (3)	0.060 (3)	0.001 (3)	0.017 (3)	0.007 (2)
C17	0.059 (4)	0.046 (3)	0.057 (3)	−0.012 (3)	0.002 (3)	−0.009 (2)
C16'	0.062 (4)	0.033 (3)	0.060 (3)	0.001 (3)	0.017 (3)	0.007 (2)
C17'	0.059 (4)	0.046 (3)	0.057 (3)	−0.012 (3)	0.002 (3)	−0.009 (2)
C18	0.081 (2)	0.0390 (19)	0.054 (2)	−0.0103 (17)	0.0197 (18)	−0.0048 (16)

### Geometric parameters (Å, °)

**Table:**

Cu1—Cl1	2.102 (3)	C9—H9	0.9300
Cu1—N1	1.928 (4)	C10—H10	0.9300
Cu1—N2	2.236 (4)	C11—C12	1.458 (4)
Cu1'—Cl1	2.112 (6)	C13—C14	1.431 (4)
Cu1'—N1	2.268 (7)	C13—C18	1.501 (4)
Cu1'—N2	1.858 (6)	C14—C15	1.504 (4)
N1—C1	1.339 (4)	C15—C16'	1.526 (7)
N1—C12	1.362 (4)	C15—C16	1.530 (5)
N2—C10	1.329 (4)	C15—H15A	0.9700
N2—C11	1.353 (4)	C15—H15B	0.9700
N3—C13	1.316 (4)	C15—H15C	0.9700
N3—C5	1.358 (3)	C15—H15D	0.9700
N4—C14	1.325 (3)	C16—C17	1.538 (6)
N4—C6	1.356 (4)	C16—H16A	0.9700
C1—C2	1.393 (4)	C16—H16B	0.9700
C1—H1	0.9300	C17—C18	1.528 (5)
C2—C3	1.361 (4)	C17—H17A	0.9700
C2—H2	0.9300	C17—H17B	0.9700
C3—C4	1.404 (4)	C16'—C17'	1.515 (8)
C3—H3	0.9300	C16'—H16C	0.9700
C4—C12	1.395 (4)	C16'—H16D	0.9700
C4—C5	1.457 (4)	C17'—C18	1.492 (7)
C5—C6	1.399 (4)	C17'—H17C	0.9700
C6—C7	1.453 (4)	C17'—H17D	0.9700
C7—C11	1.402 (4)	C18—H18A	0.9700
C7—C8	1.411 (4)	C18—H18B	0.9700
C8—C9	1.374 (4)	C18—H18C	0.9700
C8—H8	0.9300	C18—H18D	0.9700
C9—C10	1.394 (5)		
			
N1—Cu1—Cl1	155.21 (19)	N3—C13—C14	121.6 (2)
N1—Cu1—N2	80.06 (15)	N3—C13—C18	118.4 (3)
Cl1—Cu1—N2	124.71 (15)	C14—C13—C18	120.0 (2)
N2—Cu1'—Cl1	150.9 (4)	N4—C14—C13	120.8 (2)
N2—Cu1'—N1	80.7 (2)	N4—C14—C15	117.1 (3)
Cl1—Cu1'—N1	127.9 (3)	C13—C14—C15	122.1 (2)
C1—N1—C12	117.2 (3)	C14—C15—C16'	112.9 (4)
C1—N1—Cu1	124.7 (2)	C14—C15—C16	114.5 (3)
C12—N1—Cu1	118.1 (2)	C14—C15—H15A	108.6
C1—N1—Cu1'	136.7 (3)	C16—C15—H15A	108.6
C12—N1—Cu1'	106.1 (2)	C14—C15—H15B	108.6
C10—N2—C11	117.8 (3)	C16—C15—H15B	108.6
C10—N2—Cu1'	122.8 (3)	H15A—C15—H15B	107.6
C11—N2—Cu1'	119.3 (3)	C14—C15—H15C	109.0
C10—N2—Cu1	134.2 (2)	C16'—C15—H15C	109.0
C11—N2—Cu1	107.9 (2)	C14—C15—H15D	109.0
C13—N3—C5	117.9 (2)	C16'—C15—H15D	109.0
C14—N4—C6	117.7 (2)	H15C—C15—H15D	107.8
N1—C1—C2	123.2 (3)	C15—C16—C17	109.5 (3)
N1—C1—H1	118.4	C15—C16—H16A	109.8
C2—C1—H1	118.4	C17—C16—H16A	109.8
C3—C2—C1	119.4 (3)	C15—C16—H16B	109.8
C3—C2—H2	120.3	C17—C16—H16B	109.8
C1—C2—H2	120.3	H16A—C16—H16B	108.2
C2—C3—C4	119.4 (3)	C18—C17—C16	109.2 (4)
C2—C3—H3	120.3	C18—C17—H17A	109.8
C4—C3—H3	120.3	C16—C17—H17A	109.8
C12—C4—C3	117.8 (3)	C18—C17—H17B	109.8
C12—C4—C5	119.5 (3)	C16—C17—H17B	109.8
C3—C4—C5	122.7 (3)	H17A—C17—H17B	108.3
N3—C5—C6	120.7 (3)	C17'—C16'—C15	111.4 (6)
N3—C5—C4	119.0 (3)	C17'—C16'—H16C	109.3
C6—C5—C4	120.3 (3)	C15—C16'—H16C	109.3
N4—C6—C5	121.4 (3)	C17'—C16'—H16D	109.3
N4—C6—C7	118.6 (3)	C15—C16'—H16D	109.3
C5—C6—C7	120.0 (3)	H16C—C16'—H16D	108.0
C11—C7—C8	117.5 (3)	C18—C17'—C16'	113.4 (6)
C11—C7—C6	120.0 (3)	C18—C17'—H17C	108.9
C8—C7—C6	122.5 (3)	C16'—C17'—H17C	108.9
C9—C8—C7	119.4 (3)	C18—C17'—H17D	108.9
C9—C8—H8	120.3	C16'—C17'—H17D	108.9
C7—C8—H8	120.3	H17C—C17'—H17D	107.7
C8—C9—C10	118.7 (3)	C17'—C18—C13	116.0 (4)
C8—C9—H9	120.6	C13—C18—C17	112.4 (3)
C10—C9—H9	120.6	C13—C18—H18A	109.1
N2—C10—C9	123.6 (3)	C17—C18—H18A	109.1
N2—C10—H10	118.2	C13—C18—H18B	109.1
C9—C10—H10	118.2	C17—C18—H18B	109.1
N2—C11—C7	122.9 (3)	H18A—C18—H18B	107.9
N2—C11—C12	117.4 (3)	C17'—C18—H18C	108.3
C7—C11—C12	119.7 (3)	C13—C18—H18C	108.3
N1—C12—C4	123.0 (3)	C17'—C18—H18D	108.3
N1—C12—C11	116.5 (3)	C13—C18—H18D	108.3
C4—C12—C11	120.5 (3)	H18C—C18—H18D	107.4
			
N1—Cu1—Cl1—Cu1'	175.5 (12)	C7—C8—C9—C10	0.3 (5)
N2—Cu1—Cl1—Cu1'	−6.9 (7)	C11—N2—C10—C9	1.7 (5)
N2—Cu1'—Cl1—Cu1	165.8 (15)	Cu1'—N2—C10—C9	−177.9 (3)
N1—Cu1'—Cl1—Cu1	−2.1 (6)	Cu1—N2—C10—C9	−175.5 (3)
Cl1—Cu1—N1—C1	−3.3 (6)	C8—C9—C10—N2	−1.9 (5)
N2—Cu1—N1—C1	178.7 (3)	C10—N2—C11—C7	0.2 (4)
Cl1—Cu1—N1—C12	176.5 (3)	Cu1'—N2—C11—C7	179.8 (3)
N2—Cu1—N1—C12	−1.5 (2)	Cu1—N2—C11—C7	178.1 (2)
Cl1—Cu1—N1—Cu1'	−174.7 (14)	C10—N2—C11—C12	−179.7 (3)
N2—Cu1—N1—Cu1'	7.3 (10)	Cu1'—N2—C11—C12	−0.1 (4)
N2—Cu1'—N1—C1	178.5 (3)	Cu1—N2—C11—C12	−1.8 (3)
Cl1—Cu1'—N1—C1	−7.5 (6)	C8—C7—C11—N2	−1.7 (4)
N2—Cu1'—N1—C12	0.7 (3)	C6—C7—C11—N2	178.1 (3)
Cl1—Cu1'—N1—C12	174.7 (3)	C8—C7—C11—C12	178.2 (3)
N2—Cu1'—N1—Cu1	−171.3 (12)	C6—C7—C11—C12	−2.0 (4)
Cl1—Cu1'—N1—Cu1	2.8 (8)	C1—N1—C12—C4	−0.8 (4)
Cl1—Cu1'—N2—C10	9.0 (8)	Cu1—N1—C12—C4	179.4 (2)
N1—Cu1'—N2—C10	179.3 (3)	Cu1'—N1—C12—C4	177.5 (3)
Cl1—Cu1'—N2—C11	−170.6 (6)	C1—N1—C12—C11	−179.2 (3)
N1—Cu1'—N2—C11	−0.3 (3)	Cu1—N1—C12—C11	1.0 (4)
Cl1—Cu1'—N2—Cu1	−162.4 (18)	Cu1'—N1—C12—C11	−0.9 (3)
N1—Cu1'—N2—Cu1	7.9 (11)	C3—C4—C12—N1	0.4 (4)
N1—Cu1—N2—C10	179.2 (3)	C5—C4—C12—N1	180.0 (3)
Cl1—Cu1—N2—C10	0.2 (4)	C3—C4—C12—C11	178.8 (3)
N1—Cu1—N2—C11	1.8 (2)	C5—C4—C12—C11	−1.6 (4)
Cl1—Cu1—N2—C11	−177.18 (19)	N2—C11—C12—N1	0.8 (4)
N1—Cu1—N2—Cu1'	−170.7 (13)	C7—C11—C12—N1	−179.1 (3)
Cl1—Cu1—N2—Cu1'	10.3 (11)	N2—C11—C12—C4	−177.7 (3)
C12—N1—C1—C2	0.6 (5)	C7—C11—C12—C4	2.4 (4)
Cu1—N1—C1—C2	−179.6 (3)	C5—N3—C13—C14	0.8 (4)
Cu1'—N1—C1—C2	−177.0 (3)	C5—N3—C13—C18	−178.1 (3)
N1—C1—C2—C3	−0.1 (5)	C6—N4—C14—C13	1.3 (4)
C1—C2—C3—C4	−0.3 (5)	C6—N4—C14—C15	179.7 (3)
C2—C3—C4—C12	0.1 (4)	N3—C13—C14—N4	−1.4 (4)
C2—C3—C4—C5	−179.4 (3)	C18—C13—C14—N4	177.5 (3)
C13—N3—C5—C6	−0.2 (4)	N3—C13—C14—C15	−179.7 (3)
C13—N3—C5—C4	178.3 (3)	C18—C13—C14—C15	−0.8 (4)
C12—C4—C5—N3	−178.1 (3)	N4—C14—C15—C16'	−159.2 (5)
C3—C4—C5—N3	1.5 (4)	C13—C14—C15—C16'	19.2 (6)
C12—C4—C5—C6	0.5 (4)	N4—C14—C15—C16	172.1 (3)
C3—C4—C5—C6	−180.0 (3)	C13—C14—C15—C16	−9.5 (5)
C14—N4—C6—C5	−0.7 (4)	C14—C15—C16—C17	41.6 (5)
C14—N4—C6—C7	−179.1 (2)	C15—C16—C17—C18	−64.6 (5)
N3—C5—C6—N4	0.1 (4)	C14—C15—C16'—C17'	−46.3 (9)
C4—C5—C6—N4	−178.4 (2)	C16—C15—C16'—C17'	53.0 (8)
N3—C5—C6—C7	178.5 (2)	C15—C16'—C17'—C18	57.2 (11)
C4—C5—C6—C7	0.0 (4)	C16'—C17'—C18—C13	−38.7 (10)
N4—C6—C7—C11	179.2 (2)	C16'—C17'—C18—C17	51.3 (7)
C5—C6—C7—C11	0.8 (4)	N3—C13—C18—C17'	−170.6 (5)
N4—C6—C7—C8	−1.0 (4)	C14—C13—C18—C17'	10.4 (7)
C5—C6—C7—C8	−179.4 (3)	N3—C13—C18—C17	156.8 (3)
C11—C7—C8—C9	1.4 (4)	C14—C13—C18—C17	−22.1 (5)
C6—C7—C8—C9	−178.4 (3)	C16—C17—C18—C13	54.5 (5)
